# Therapeutic Activities of Multipotent Stromal Cells for Islet Regeneration

**DOI:** 10.3390/cells15060488

**Published:** 2026-03-10

**Authors:** Nazihah Rasiwala, Gillian I. Bell, Nouran N. Al-Banaa, David A. Hess

**Affiliations:** 1 Department of Physiology and Pharmacology, Schulich School of Medicine and Dentistry, Western University, London, ON N6A 3K7, Canada; 2 Molecular Medicine Research Laboratories, Robarts Research Institute, London, ON N6A 3K7, Canada

**Keywords:** islets, diabetes, beta cell regeneration, multipotent stromal cells, mesenchymal stem cells

## Abstract

Diabetes mellitus is a global healthcare issue of epidemic proportions. At the root of these disorders, characterized by poor glucose regulation and insulin deficiencies, is the pancreatic beta cell and insufficient insulin signal transduction in peripheral tissues. Residual c-peptide secretion and persisting beta cells have been found in patients who have been living with type 1 diabetes for over 50 years. Thus, beta cell regeneration has been vastly studied in rodents, and many agents to expand beta cell mass are under rigorous investigation for the treatment of diabetes. Multipotent stromal cells (MSC), isolated from human bone marrow, have an immunomodulatory and pro-regenerative secretome that can aid in repairing damaged tissues, including pancreatic islets. MSC transplantation has been shown to reduce hyperglycemia and orchestrate islet repair in experimental diabetes models and is currently being assessed in clinical trials. While the immunomodulatory mechanisms of MSC are well-studied, the beta-cell-regenerative mechanisms are unknown. MSC likely play a regenerative role by signaling to resident progenitor or precursor cells in the pancreas; however, the decades-long controversy surrounding the origin of regenerated adult beta cells remains unresolved. Herein, we take a deep dive into the role of MSC in the treatment of diabetes and the potential cellular mechanisms behind the MSC stimulation of beta cell regeneration.

## 1. Diabetes Mellitus: Pathology, Complications, Current Treatments

Diabetes mellitus is a group of metabolic disorders characterized by loss of blood glucose homeostasis resulting in hyperglycemia. Type 1 diabetes (T1D) is a chronic, autoimmune disorder caused by immune cell-mediated destruction of insulin-producing pancreatic beta cells. Type 2 diabetes mellitus (T2D) evolves from insulin resistance, an inability of peripheral tissues to respond to insulin and uptake glucose, with diminishing insulin secretion by beta cells over time [1]. Acute complications of diabetes include ketoacidosis and non-ketotic hyperosmolar coma. Chronic complications include carotid, coronary, and peripheral artery disease, kidney dysfunction, neuropathy, and retinopathy. Long-standing diabetes is also a risk factor for cognitive decline in later life [2].

Exogenous insulin therapy is the cornerstone of diabetes treatment for T1D and late-stage T2D. While there have been many advancements made in insulin therapy since its inception in 1921, including the development of ultralong insulin analogues and glucose-responsive insulin patch-pump systems [3], monotherapy often fails to address the broader metabolic and cardiovascular disturbances associated with diabetes. Consequently, several non-insulin pharmacologic agents such as metformin, sodium-glucose co-transporters-2 (SGLT2) inhibitors, dipeptidyl-peptidase 4 (DPP-4) inhibitors, glucagon-like peptide-1 (GLP-1) receptor agonists, and amylin analogues have been administered alongside insulin to optimize glycemic control and reduce secondary complications [4]. To restore insulin secretory mechanisms, replacement or regeneration of beta cells themselves, alongside protection from ongoing beta cell depletion, represent leading approaches to cure diabetes [3].

## 2. Evidence of Beta Cell Regeneration in Humans

During peri-natal development, beta cell proliferation peaks within the first 2 years of life and then gradually declines. A pool of proliferating beta cells persists into adulthood; however, these cells are rare (<0.5%) [5]. Rapid increases in beta cell mass can occur during pregnancy and obesity to respond to increased metabolic demand and illustrate the capacity of adult human islets to regenerate when physiologically required [6,7].

One of the most compelling demonstrations of endogenous beta cell regeneration comes from the 2010 Joslin Medalist Study, which revealed residual beta cell function in individuals with long-standing T1D (>50 years). Among 411 participants, 67.4% had detectable C-peptide levels, indicating ongoing production of insulin despite long-term autoimmunity [8]. Furthermore, histological analysis of pancreas tissue from a subset of participants (N = 9) revealed the presence of insulin-positive cells, appearing as singlets or small islet-like clusters, irrespective of circulating c-peptide levels [8]. More recent evidence provided by Lam et al. in a study of 47 post-mortem T1D pancreata showed 64% had residual beta cells, and a few samples from both recent-onset and long-standing T1D individuals had widespread insulin+ islets [9]. These findings suggested that steady-state beta cell turnover occurred during T1D despite chronic autoimmune destruction.

Recapitulating human beta cell development in utero by neogenesis from ductal cells has been proposed as a mechanism by which beta cell regeneration occurs. Insulin+ ductal cells have been observed in obese individuals [10] and pregnant women [6]. A study of 10 recent onset T1D pancreata showed a marked increase in proliferating alpha and beta cells compared to controls, particularly in islets with insulitis [11]. Thus, therapies that shift the balance towards regeneration versus destruction of beta cells may represent a promising strategy towards a curative therapy for diabetes.

## 3. Multipotent Stromal Cells

Multipotent stromal cells (MSC), also referred to as mesenchymal stem cells, are mesodermal progenitors distributed primarily in the perivascular space in many tissues [12]. The International Society for Cell and Gene Therapy defines MSC as having three distinct characteristics: (1) cell growth adherent to plastic in culture, (2) expression of the stromal markers CD73, CD90, and CD105 on at least 95% of cells without meaningful expression of hematopoietic markers, and (3) the potential to differentiate into bone, cartilage, and fat in vitro [13]. Lineage tracing and single-cell RNA sequencing have revealed that MSC are heterogeneous in phenotype and function and distinct types of MSC exist even within the same tissue [14]. MSC from many tissues, including bone marrow (BM), umbilical cord (UC), adipose (AD), and the pancreas (PANC), have been isolated and studied for regenerative therapy applications.

### 3.1. MSC Production of Bioactive Stimuli

The expansive clinical applications of MSC originate from their ability to modulate the local tissue environment via paracrine signaling of diverse bioactive stimuli [14]. Multifaceted actions on tissue regeneration, immunomodulation, stem and progenitor cell differentiation, and vascularization have been demonstrated by MSC in vivo [15]. As such, MSC act as the “paramedics of the body,” [14] and their perivascular location is advantageous for sensing and homing to areas of tissue damage to dampen inflammation and coordinate a reparative response. MSC response depends on the type of damage presented, pre-existing inflammatory status, and the regenerative capacity of the tissue.

The excitement for MSC use as a therapeutic cell type is accentuated by the potential to modulate both innate and adaptive immune responses. MSC exert potent immunomodulatory effects that can mitigate the autoimmune pathology of T1D by both suppressing pathogenic immune responses and promoting inflammation resolution. An array of studies have shown that MSC inhibit adaptive T- and B-cell expansion, reduce inflammatory cytokine secretion, and promote the differentiation of T-regulatory cells (Tregs) in the presence of inflammatory stimuli such as IFNg [16,17,18,19,20]. While MSC can modulate immune responses through cell-to-cell contact and by cell surface expression of the negative costimulatory molecule B7-H4 [19,20,21], immunomodulatory action is also elicited, in part, via the production of indoleamine 2,3-dioxygenase (IDO), prostaglandin E2, TGF-beta, and interleukin-10 (IL-10), leading to the inhibition of effector T-cell proliferation, reduction in Th1/Th17 polarization, and induction of Tregs that maintain immune tolerance to β-cell antigens [22,23]. In addition, MSC secrete factors such as hepatocyte growth factor (HGF) and TGF-β that promote resolution of inflammation and facilitate regeneration of the islet microenvironment [24,25]. MSC also dampen innate immunity by inhibiting the maturation and antigen-presentation function of dendritic cells [26], as well as downregulating the cytotoxicity of NK cells and tissue resident macrophages [21]. MSC skew macrophage and dendritic cell phenotypes toward anti-inflammatory states, which contributes to dampening autoreactive T-cell activation and enables tissue repair [27,28].

**Figure 1:**
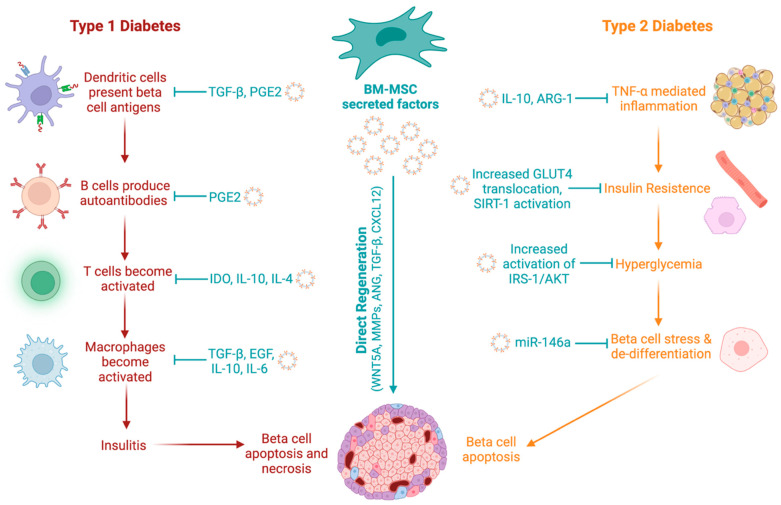
Pleiotropic mechanisms by which the secretory activities of MSC impact diabetes. In T1D, autoimmune destruction of beta cells is mediated by adaptive and innate immune cell types. In T2D, hyperglycemia results from insulin resistance with associated inflammation. Increased inflammation leads to beta-cell stress, dedifferentiation, and apoptosis. MSC secreted factors can dampen inflammation and insulitis during T1D and preserve beta cell identity in T2D. Created with Biorender.com.

These combined immunomodulatory and regenerative properties position MSC as a promising candidate for both T1D and T2D therapy by rebalancing immune responses, supporting long-term inflammation resolution, and stimulating beta cell expansion and maturation while protecting residual β cells from immune-mediated destruction (Figure 1).

#### 3.1.1. MSC-Induced Islet Regeneration in Animal Models of T1D

Hyperglycemia can be chemically induced in rodents through the administration of streptozotocin (STZ), a beta-cell-specific toxin. Hess et al., in 2003 [29], found that hyperglycemia in non-obese diabetic/severe combined immunodeficiency (NOD/SCID) mice treated with multiple low-dose STZ could be resolved by bone marrow transplantation. Specifically, intravenous injection of green fluorescent protein (GFP) labeled mouse bone marrow cells and purified c-kit+ hematopoietic stem/progenitor cells reduced blood glucose, increased serum insulin, and increased the number of insulin+ islets over a one-month period. Transplanted GFP+ bone marrow cells did not differentiate into beta cells, but surrounded damaged islets and stimulated endogenous islet regeneration [29]. These results were later confirmed using progenitor cells from human BM and umbilical cord blood [30].

In 2006, Lee et al. found that human BM-MSC delivered via intracardiac infusion into STZ-treated NOD/SCID mice reduced blood glucose, increased serum insulin, and recovered islet number [31]. Similarly, BM-MSC transplantation in combination with exendin-4, a long-acting GLP-1R agonist, normalized fasting blood glucose levels, increased beta cell mass and maturity, and restored beta cell function in STZ-treated Sprague Dawley rats over a one-month period [32].

In 2012, Bell et al. established that tail vein injection of human BM-MSC could ameliorate hyperglycemia, augment systemic insulin release, and increase beta cell mass; however, regenerative induction showed extreme donor-dependent variability [30,33]. When delivered systemically, MSC were difficult to locate in the pancreas [30], likely due to MSC first lodging in the lung micro-vasculature [34]. Liu et al. also showed that fluorescently labeled UC-MSC IV-injected into STZ-treated C57BL/6J enhanced viability, insulin secretion, and beta cell function while attenuating beta cell injury by protecting the beta cells from high glucose-induced oxidative stress. The UC-MSC were detectable in the pancreas, liver, lung, spleen and kidney at 24 h after injection; however, detection was gradually reduced over 7 days [35]. Thus, it was proposed that local delivery of MSC may improve survival and islet-regenerative function. In 2012, Bell et al. established intra-pancreatic (iPAN) injection as a successful method to deliver stem cells directly to the site of injury, where they can more effectively induce tissue regeneration [36]. Several groups have shown iPAN injection as being a superior delivery method for MSC compared to IV injection. The iPAN injection of human BM-MSC into STZ-treated C57BL/6 mice showed that the injected cells survived in the pancreas for up to 1 month, and that the transplanted mice had reduced blood glucose and improved plasma insulin and islet morphology [37]. The iPAN-injected mice also had modulated macrophage number and activation state. In comparison, IV-injected cells localized primarily to the lungs and did not demonstrate these beneficial effects [37]. Similarly, Khatri et al. assessed engraftment of human telomerase reverse transcriptase (hERT) MSC following IV- or iPAN-injection into mice that had undergone partial pancreatectomy [38]. They showed that all iPAN-injected mice had MSC detectable in the pancreas 8 days after injection, with no detection in other organs. However, four out of eight IV-injected mice had MSC in the lungs, while only one of eight had detectable cells in the pancreas. Only iPAN-injected hTERT-MSC stimulated growth factors, anti-inflammatory markers, and EGF expression in the pancreas, leading to recovery from pancreatic injury [38]. More recently, the iPAN injection of 500,000 human AD-MSC further decreased blood glucose levels and increased islet number over a 30-day period in STZ-treated mice compared to IV injection [39]. iPAN transplantation of AD-MSC increased the transcription of anti-inflammatory cytokines EGF and IL-10, but not pro-inflammatory cytokines IL-1b or TNF-a in the pancreas [39]. Notably, MSC native to the human pancreas were isolated as vimentin+/nestin+ plastic adherent cells when culturing human islets. The iPAN-injection of concentrated conditioned media and exosomes from PANC-MSC reduced hyperglycemia in STZ-treated mice [40,41].

The non-obese diabetic (NOD) mouse spontaneously develops autoimmune diabetes, and the mechanisms of insulitis are similar to those of human T1D. Madec et al. demonstrated that murine MSC could prevent autoimmune beta cell destruction when injected intraperitoneally or intravenously into four-week-old female NOD mice via the induction of regulatory T cells [42]. Systemic delivery of human AD-MSC also lowered the incidence of programmed cell death receptor ligand 1 antibody-induced diabetes and resulted in improved blood glucose levels, preserved islet area and insulin content, and prevented CD3+ T cell and macrophage accumulation in islets [34]. Therefore, MSC play an important role in beta cell protection in autoimmune diabetes (Figure 1). However, to date, there has primarily been a failure to translate success in NOD models to the prevention of T1D in humans. This is due to NOD mice having a more aggressive disease onset than humans, as well as the comparably large genetic diversity in human T1D. Technologies to edit the NOD genome to produce NOD-derived recombinant congenic strains for T1D research can provide advantages in mimicking the genetic variation among patients, but also require substantial time and resources [43]. A powerful mouse model to study “human” T1D would be to reconstitute a mouse with a T1D-prone human immune system that targets beta cells [44].

#### 3.1.2. MSC-Induced Islet Regeneration in Animal Models of T2DM

Rodent models have also been used to assess the effects of MSC on insulin resistance and insulin signaling. T2D-like conditions can be modelled in rats using a high-fat diet and STZ. Infusion of MSC at one week and three weeks post-STZ injection lowered blood glucose for a 2–3-week period in T2D-like rats [45]. Furthermore, MSC treatment improved insulin sensitivity assessed via a hyperinsulinemic-euglycemic clamp and restored membrane concentrations of GLUT4 in skeletal muscle and adipose tissue via activation of insulin receptor substrate-1 [45]. Interestingly, the delivery of secreted exosomes from UC-MSC similarly improved insulin sensitivity, promoted GLUT4 translocation in muscle tissue, and reduced STZ-induced beta cell apoptosis [46]. Thus, MSC can modify multiple pathological processes underlying T2D (Figure 1).

### 3.2. Identifying Regenerative Proteins Secreted by MSC

Human MSC secrete a variety of growth factors, chemokines, and cytokines; however, this secretome appears to vary significantly, depending on the donor’s health, inflammatory status, and the niche where the MSC reside [40,47]. Notably, BM-MSC show extreme donor-dependent variability in the induction of islet regeneration in STZ-treated NOD/SCID mice [30,48,49]. Studies from Bell et al. found that ~25% of human BM-MSC samples could significantly reduce glycemia (highly regenerative), whereas ~33% of samples modestly reduced glycemia (moderately regenerative), and ~42% did not reduce glycemia at all (non-regenerative) [33]. While surface marker expression is important to identify that MSC are indeed the cell type being studied, surface marker expression alone cannot shed light on their regenerative potential. The secretory profile of the MSC is what will determine whether they are able to support regeneration, and different injury/disease models will likely require unique secretory profiles for optimal regeneration. Kuljanin et al. showed that the most compelling donor characteristic in determining whether MSC will be regenerative or not, at least in the context of beta cell regeneration, is donor BMI [49]. MSC from 80% of BM donors with “normal” BMI (<25 kg/m^2^) had regenerative secretory profiles. Importantly, MSC from BM donors with BMI in the overweight or obese range were all classified as having non-regenerative secretory profiles [49]. It is unclear if using donor BMI as a pre-screening tool to determine the probability of regenerative capacity is consistent across MSC sources (UC, PANC, AD); however, donor health is likely to play a critical role in the quality of the expanded MSC and their secretome.

Quantification of global mRNA expression in highly regenerative vs. non-regenerative BM-MSC samples revealed that highly regenerative BM-MSC consistently upregulated transcription of effectors downstream of canonical Wnt/beta-catenin signaling [33], indicating this pathway was activated in highly regenerative MSC. Quantitative mass-spectrometry-based proteomics further revealed that members of the Wnt-signaling pathway, such as Wnt1-inducible-signalling protein (WISP) 2, Wnt5a, Wnt5b, Spondin-2, and secreted frizzled-related protein 1, were consistently increased in highly regenerative MSC compared to non-regenerative MSC [47,49]. Thus, BM-MSC samples could be accurately categorized as regenerative or non-regenerative based on predictive proteomic signatures [49].

Wnt/beta-catenin signaling can be modulated in BM-MSC by the addition of glycogen synthase kinase-3 (GSK3) inhibitor CHIR99021 during culture [47]. GSK3 is an intracellular kinase and part of the beta-catenin destruction complex that acts to phosphorylate beta-catenin destined for degradation [50]. Inhibition of GSK3 increases intracellular accumulation and nuclear translocation of beta-catenin, resulting in the transcription of downstream Wnt-pathway effectors. Compared to unmodified BM-MSC, MSC treated with CHIR99021 showed increased intracellular beta-catenin accumulation [47] and nucleus-localized beta-catenin [51]. Moreover, the expression of *BCL9* and *MYC* genes transcribed during active Wnt-signaling was similarly increased via treatment of BM-MSC with CHIR99021 [52].

In 2019, Kuljanin et al. assessed the iPAN injection of conditioned media (CdM) generated by BM-MSC treated with CHIR99021 (Wnt+ CdM) in multiple low-dose STZ-treated NOD/SCID mice [52]. Compared to the iPAN injection of unstimulated control CdM, Wnt+ CdM injection consistently reduced non-fasted blood glucose, increased serum insulin levels, and improved glucose tolerance in mice at one-month post-injection. Remarkably, islet number, beta cell mass, beta cell proliferation, and expression of beta cell maturation markers Nkx6.1 and MafA were increased in the pancreas of mice treated with Wnt+ CdM compared to controls [52]. While this work demonstrated that Wnt+ CdM could induce beta cell regeneration and maturation without MSC transfer, the potential cell types in the pancreas that respond to regenerative signals and the cellular mechanisms driving beta cell regeneration remained unclear.

## 4. Potential ‘Signal-Receiving’ Cells in the Pancreas

**Figure 2:**
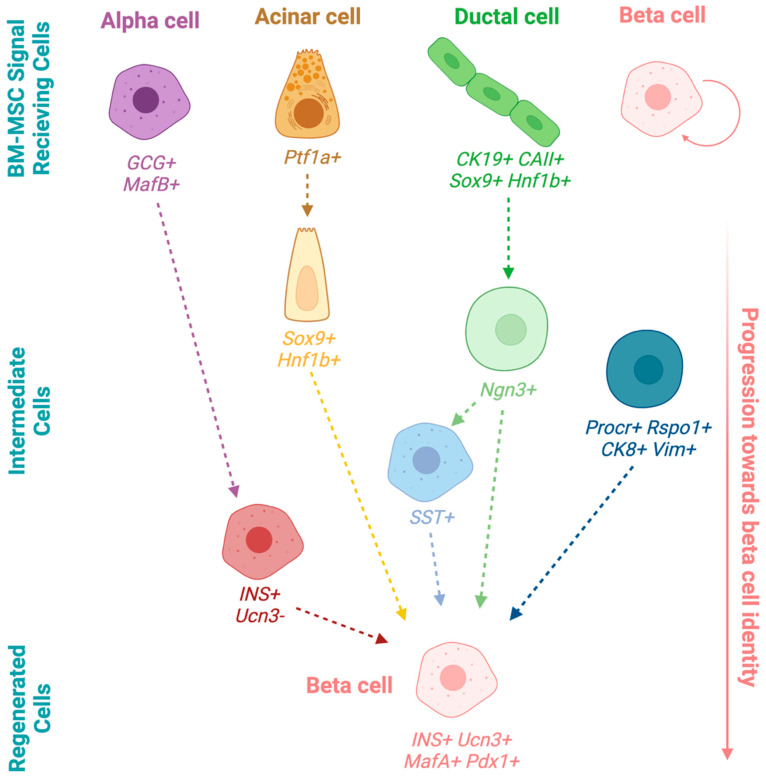
Overview of pancreas cell types that progress towards regenerating beta cells. Alpha cells transition to beta cells via a Ucn3-negative immature beta cell intermediate. Acinar-to-beta cell transition via a Sox9+/Hnf1b+ intermediate. Ductal cells may regain expression of Ngn3+ and convert to beta cells. Procr+ cells may directly produce beta cells that undergo self-replication. Created with Biorender.com.

MSC modify their surrounding environment by delivering apoptotic, proliferative, or differentiative factors to nearby cells. Communication with tissue-resident progenitor cells also occurs during times of damage when tissue remodeling is required [14]. Thus, islet preservation demonstrated by BM-MSC in experimental models of T1D and T2D may occur via the activation of multiple cell types in the pancreas (Figure 2). Whether an insulin-negative beta cell precursor exists in the adult human pancreas remains a controversial query. Elegant genetic lineage tracing studies have allowed tracking of various cell lineages in the pancreas to assess their contribution to beta-like cell populations. However, due to variability in experimental models and methodologies used during lineage tracing, the data to date have not converged on a prevailing signal-receiving cell type in the pancreas as the source of regenerated beta cells (Table 1). Multiple pancreatic resident cell types that respond to islet regenerative cues have been proposed (Figure 2).

### 4.1. Potential Mechanisms of Beta Cell Regeneration

Multiple regenerative mechanisms contribute to the formation of new beta cells. Competing viewpoints on beta cell regeneration in situ include: (1) Proliferation: pre-existing beta cells undergo self-replication to form new beta cells. (2) Transdifferentiation: new beta cells are formed by conversion from a terminally differentiated non-insulin, pancreatic cell type, or (3) Neogenesis: new beta cells are formed by differentiation from facultative endocrine progenitor cells. Likely, each mechanism may occur simultaneously, depending on the modality of pancreas damage employed.

### 4.2. Beta Cell Proliferation

A stem-cell niche, such as those found in highly regenerative tissues like the intestinal tract, hematopoietic system, or skin, has not been identified in the adult pancreas [72]. Several studies have provided evidence that a primary source of new beta cells is pre-existing beta cell proliferation in adult mice under various conditions (Table 1). Under steady-state conditions, beta cell proliferation is minimal. In a seminal study using an insulin receptor (RIP)-CreERT mouse model, Dor et al. found that the frequency of labelled beta cells did not change during 1 year of adult life, indicating that new beta cells were derived by the replication of pre-existing beta cells. An increase in beta cell proliferation following 70% pancreatectomy (Px) also indicated that regenerated beta cells were primarily formed by self-duplication in this model [53]. These findings were supported by Teta et al., who used DNA analog incorporation at the single-cell level to show that adult beta cells expanded primarily through self-duplication [73]. Using a super-efficiency, dual-recombinase-mediated genetic lineage tracing model that labelled >99% of beta cells, Zhao et al. found labelled beta cells were not diluted by the contribution of non-beta cells over a 25-week period, indicating that beta cell replication was the main source of new beta cells during homeostasis. Only following extreme beta cell loss mediated by diphtheria toxin-mediated genetic ablation did alpha and delta cell lineages contribute to the replacement of beta cells [7].

Following pancreatic damage, MSC can stimulate a beta-cell proliferative regenerative program. Khatri et al. showed that following 50% pancreatectomy, nude mice that received MSC transplant either via intravenous or intrapancreatic administration, had significantly increased BrdU incorporation in beta cells compared to controls within 8 days [38]. Intrapancreatic injection of CdM from CHIR99021-treated MSC into STZ-treated NOD/SCID mice similarly increased beta cell proliferation 4 days following administration [52]. A cocktail of eight recombinant human proteins (8P), shown to consistently be increased in the secretome of regenerative MSC, also stimulated beta cell proliferation following intrapancreatic injection into STZ-treated NOD/SCID mice [74]. Therefore, MSC-induced beta cell regeneration is enabled at least in part through an endogenous beta cell proliferative mechanism.

#### 4.2.1. Alpha-to-Beta Cell Conversion

Other endocrine cells have been suggested as a possible source that can transition to replace beta cells lost during diabetes (Figure 2). Beta, alpha, delta, epsilon, and pancreatic polypeptide cells share a common Ngn3+ multipotent progenitor during embryonic development. These mature endocrine cell types have similar transcriptomes and epigenetic patterns that allow for remarkable plasticity between islet cell types [75]. Treatment with a histone methyltransferase inhibitor in cultured islets resulted in the co-localization of glucagon and insulin double-positive cells [75]. Intraductal viral delivery of beta cell factors Pdx1 and MafA in alloxan-treated mice reversed hyperglycemia and permitted alpha cell reprogramming into beta cells [54]. Ectopic Pax4 expression and Arx inactivation in alpha cells in vivo also induced alpha to beta cell conversion [55,56]. GABA treatment in STZ-treated mice showed a modest increase in insulin+ cells originating from traced glucagon+ cells [57]. Interestingly, residual islets from donors with established T1D for >4 years showed glucagon+ cells that lacked Arx expression and co-expressed beta cell markers Pdx1 and Nkx6.1 [76], suggesting alpha-to-beta cell conversion may occur during T1D.

Beta cell emergence from an alpha cell origin has been traced using multiple models of beta cell ablation (Table 1). Lineage tracing of beta cells following diphtheria toxin-mediated ablation (>99%) showed a ten-fold dilution in beta cell labelling as beta cell mass tripled. Subsequent glucagon+ cell tracing showed a 65% contribution of alpha cells to the regenerated beta cells at 1 month after ablation [58]. Mechanistically, alpha cells contribute to beta cell regeneration via an immature beta-like intermediate that expresses insulin but not beta cell maturation marker urocortin 3 [59]. In human neonatal islets, Urocortin3-negative beta cells are observed at the islet periphery in T1D pancreata [59] and may represent either regenerating or dedifferentiating beta cells.

While there is substantial evidence that alpha cells can be converted to beta cells, whether MSC or their secreted factors can activate this transdifferentiation remains under investigation. Intrapancreatic injection of MSC-CdM into STZ-treated mice induced islet regeneration with attributes consistent with the induction of alpha-to-beta cell transition [52]. Within 24 h of injection, there was an increase in glucagon+/insulin+ co-expressing cells detected. The alpha cell frequency was also decreased alongside an increase in beta cell frequency [52]. However, the use of alpha cell lineage tracing models is essential to definitively assess if alpha cells have contributed to the recovered beta cell population. Unpublished data (Sharma et al. Manuscript in preparation) from our lab in STZ-treated Glucagon-iCre; Rosa26-TdTomato (GcgxTdT) mice demonstrated that STZ administration alone led to significant conversion of alpha cells to beta cells that was further enhanced within 4 days of intrapancreatic administration of CHIR99021-treated-MSC CdM. Identifying the MSC-secreted molecules that can stimulate alpha-to-beta cell conversion will be an important step forward in understanding how to harness this regenerative mechanism as a treatment for diabetes.

#### 4.2.2. Delta-to-Beta Cell Conversion

Delta cells have also been proposed as a beta cell precursor whose differentiative capacity may be able to be initiated by MSC-secreted factors to replenish beta cells in diabetes (Figure 2). Somatostatin+ cell lineage tracing in juvenile mice has shown that delta cells can reconstitute beta cells via an Ngn3+ intermediate following diphtheria toxin-induced injury [70]. Pax4-overexpressing somatostatin+ delta cells in the gastrointestinal tract have also recently been traced to form GI-resident beta-like cells that express insulin in vivo and secrete insulin in response to glucose in vitro [77].

#### 4.2.3. Acinar-to-Beta Cell Conversion

The pancreas is comprised of >95% exocrine tissue and <5% endocrine tissue [78]. The abundance of acinar cells makes them an attractive source to replace lost beta cells (Figure 2). In 2008, Zhou et al. demonstrated that acinar cells could be reprogrammed to beta cells upon viral delivery of beta cell genes *Pdx1, MafA*, and *Ngn3* directly to the pancreas [61]. Ectopic expression of these three genes recovered hyperglycemia in STZ-treated mice [61]. In a follow-up study, enhanced reprogramming efficiency with the same three genes allowed regenerated beta cells to form islet-like structures over a 13-month period via induction of an acinar-to-beta DNA methylation pattern that suppressed acinar genes alongside enhanced expression of beta cell genes [79]. Acinar lineage tracing using the Pft1a promoter showed that, following pancreatic duct ligation (PDL) or a combination of PDL with STZ, acinar cells gained expression of multipotency factors Sox9 and Hnf1b and regenerated both ductal and endocrine cells, including mature insulin+ beta cells [60]. Following adenoviral delivery of *Mafa, Pdx1*, and *Ngn3* in acinar cells, scRNA-seq was performed over 10 days to demonstrate the ability of acinar cells to transdifferentiate into insulin-producing beta cells [80]. More recently, it was also shown that the inhibition of the kinase activity of focal adhesion kinase can convert acinar cells into insulin-producing cells to improve glucose homeostasis in diabetic mice [81]. While acinar cells can be stimulated to transdifferentiate into beta cells, whether MSC-secreted cytokines can initiate this process has not been determined.

### 4.3. Neogenesis from Pancreas-Resident Progenitor Cells

During fetal pancreas development, islet neogenesis occurs from a ductal cell origin (Figure 2) with fetal beta cells first derived from the differentiation of cytokeratin 19 (CK19) expressing ductal cells [82]. Fetal CK19+ ductal cells form outgrowths of insulin+ islet buds over 3–4 weeks in culture [83]. Furthermore, pancreas biopsies from nine pancreas-kidney transplant recipients with T1D demonstrated CK19+/insulin+ cells in pancreatic ducts [84], indicating ductal contribution to human beta cell regeneration in an autoimmune setting.

Prior to the development of lineage tracing modalities, evidence for a ductal-to-beta cell neogenic mechanism in the adult pancreas was limited to the detection of insulin-positive cells within the ductal epithelium [85,86]. Early studies using rodent models of 90% Px [86,87] and PDL [85] found that injury-induced ductal cell hyperplasia followed by the emergence of hormone+ cells among ducts. Diphtheria toxin-induced deletion of acinar and endocrine cells in adult mice resulted in ductal expression of Pdx1 and subsequent contribution to both acinar and endocrine cells [88]. Finally, Sox9+ centroacinar cells, positioned at the interface of acini and small ducts, could generate endocrine cells when injected into cultured human fetal pancreatic buds [89].

In 2008, back-to-back lineage tracing studies after PDL showed that ductal cells frequently gave rise to beta cells [62,90]. Since then, a wealth of lineage tracing studies using ductal markers such as CK19, carbonic anhydrase II, Sox9, Hnf1b, and Ngn3 have yielded inconsistent results as to whether ductal cells meaningfully recapitulate beta cell neogenesis in the adult mouse pancreas (Table 1). Sox9 genetic lineage tracing elegantly showed neogenic islet formation from ducts following 50% pancreatectomy [63]. Although Kopp et al. traced Sox9+ ductal cells and found no contribution to insulin+ cells following PDL [64], Sox9+ ductal cells directly contributed to beta cell neogenesis triggered by administration of EGF and gastrin following alloxan-induced beta cell ablation [65]. In Hnf1b-CreERT mice, ductal cells contributed to insulin+ cell expansion during homeostasis at a rate of 0.66% per week [67].

During development, transient expression of Ngn3 is necessary for the determination of an endocrine fate from exocrine/endocrine bipotent progenitor cells [91]. Following PDL in the adult mouse pancreas, Ngn3 activation in the ducts has been shown to be critical for beta cell mass expansion [92]. Thus, many ductal lineage tracing studies have utilized the Ngn3 promoter to elucidate the developmental mechanism of beta cell neogenesis from ducts (Table 1). During *Pax4* overexpression-mediated alpha-to-beta cell conversion [55], ductal cells re-expressed Ngn3, and these Ngn3+ cells repopulated lost alpha cells [68]. Recently, Ngn3 lineage tracing in an AKITA (insulin misfolding phenotype) diabetic mouse revealed a substantial increase in traced insulin+ and somatostatin+ cells located near ducts. Single-cell transcriptomics on islets containing traced Ngn3+ cells demonstrated that Ngn3+ cells contributed to the insulin+ cell population via a somatostatin+ intermediate [67].

Common markers that represent stem or progenitor cell populations in other tissues have also been used in the search for a facultative pancreatic progenitor cell. Recently, protein C receptor (Procr) positive cells were identified as a resident multipotent endocrine progenitor cell in the adult mouse pancreas [71] (Figure 2). Lineage tracing showed that Procr+ cells were the progeny of Ngn3+ endocrine progenitors and demonstrated multipotency by generation of four endocrine cell types in adult mice. Procr+ cells were also used to generate islet organoids that had glucose-lowering capacity after implantation in vivo [71]. Cells expressing double cortin-like kinase 1 (Dclk1) have also been suggested in mouse and human to be a quiescent, long-lived progenitor required for pancreas regeneration following injury [93].

MSC can stimulate a neogenic beta cell regenerative mechanism following pancreatic injury. Following IV transplantation of MSC into STZ-treated NOD/SCID mice, there is an increase in small islet clusters associated with the ductal epithelium, suggesting neogenesis from a ductal resident progenitor [30]. Similarly, following the iPan injection of MSC CdM, there is an increase in the association of islets with ducts [52]. In 2025, Rasiwala et al. demonstrated that a CK19+ ductal or acinar precursor contributed to islet regeneration following iPAN-injection of Wnt+ MSC CdM in STZ-treated CK19-CreERT Rosa26-mTomato mice [51]. In this lineage tracing model developed by Means et al. [94], iPAN injection of Wnt+ MSC CdM led to lasting blood glucose reduction, improved glucose tolerance, increased beta cell mass, and the frequency of TdTomato+/Insulin+ cells was increased five-fold [51]. Identifying which MSC-secreted factors drive beta-cell neogenesis from pancreatic ducts will deepen our understanding of this mechanism and how it could be effectively harnessed as a therapeutic strategy for diabetes.

### 4.4. Supportive Role of MSC in Islet Transplantation

**Figure 3:**
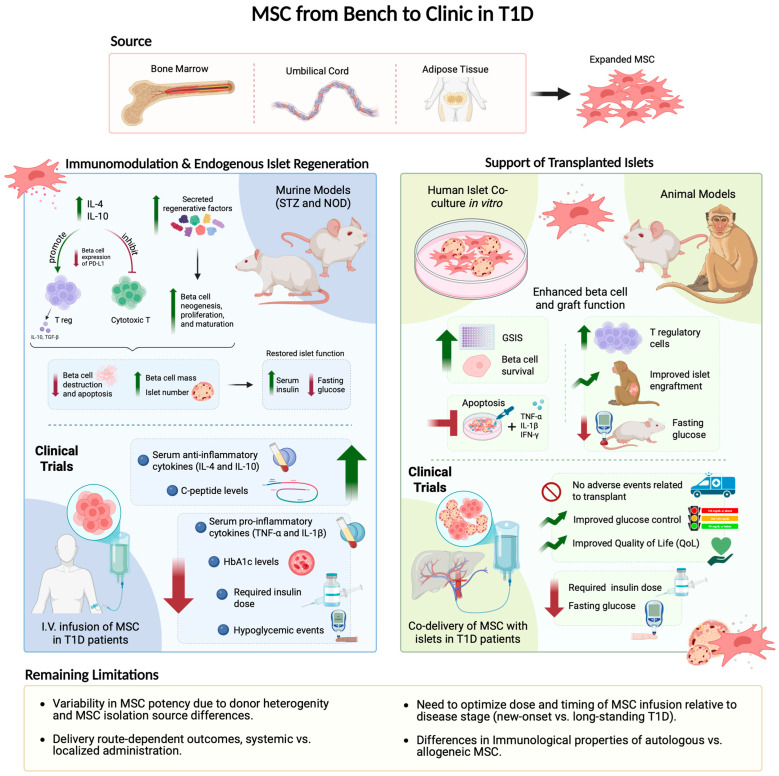
Summary of the therapeutic roles of MSC in T1D. MSC derived from bone marrow, umbilical cord, or adipose tissue have been assessed using multiple preclinical models of diabetes, where they demonstrate both immunomodulatory activity and support of endogenous islet regeneration. In addition, MSC can enhance engraftment, survival, and function of transplanted islets in replacement strategies. These preclinical findings have motivated several early-phase clinical trials focused on preservation of beta-cell function in newly diagnosed T1D. Clinical evaluation of MSC as an adjunct to islet replacement remains less developed. Results to-date support the safety and efficacy of MSC therapy. However, key limitations, including donor and source heterogeneity, route of administration, and the need for optimized dosing and timing, continue to challenge translation into the clinic. Created with Biorender.com.

During islet transplantation, poor engraftment, subpar vascularization, and chronic inflammation negatively affect implanted islets and can lead to graft failure. In islet transplantation, MSC have been used to stimulate angiogenesis [95] and improve engraftment [96,97]. In co-culture, MSC consistently facilitated beta cell survival, improved glucose-stimulated insulin secretion (GSIS), and prevented apoptosis of beta cells in the presence of pro-inflammatory cytokines IL-1b, TNFa, and IFNg [96,97,98]. Conditioned media generated from BM-MSC increased the number of beta cells, proportion of live beta cells, and proportion of proliferating beta cells in human islet cultures [41]. Transplantation of human AD-MSC with islets allowed for quicker blood glucose normalization and improved engraftment in STZ-treated immunodeficient mice compared to transplanted islets alone [96]. In NOD mice, co-encapsulation of UC-MSC and islet cells in sodium alginate reduced blood glucose levels and increased the number of T regulatory cells [99]. Finally, portal vein infusion of allogeneic islets along with autologous BM-MSC in STZ-treated cynomolgus monkeys showed that MSC co-transplantation enhanced islet engraftment, survival, and glucose-stimulated insulin release [100]. A meta-analysis of 20 islet/MSC co-culture studies found that islet cell viability and glucose responsiveness were consistently improved, although improved viability was highest for islets co-cultured indirectly with MSC rather than with direct physical contact [98]. Collectively, these studies demonstrate that MSC possess tremendous potential to support the function and survival of human islets and may be very valuable to support islet-transplantation in diabetic patients (Figure 3).

## 5. Clinical Trials Using MSC-Based Therapies for Diabetes

Human MSC are widely used in clinical trials due to: (1) ease of isolation, (2) extensive proliferative capacity in culture, (3) plentiful secretion of immunomodulatory and regenerative factors, (4) low immunogenicity, and (5) established safety profile [101]. According to the U.S. National Institute of Health ClinicalTrials.gov database, 617 clinical trials involving MSC have been recruiting, active, or completed between 2015 and 2025 [102]. Many of these trials were for diseases of the muscle, bone, and cartilage, neurological disorders, COVID-19, and cardiovascular diseases. MSC have shown a remarkable safety profile when transplanted in patients, despite variability in source tissue, donor characteristics, expansion ex vivo, and cryopreservation. MSC are also currently under investigation for the treatment of several autoimmune diseases, including diabetes, where the focus is on their ability to dampen the host immune response [103,104].

Meta-analysis of 8 MSC-based clinical trials for T1D and 5 trials for T2D suggested that MSC have an islet-protective effect in T2D, but the extent of glycemic control achieved by MSC transplantation in T1D remains inconsistent [105]. The first randomized controlled trial using MSC to intervene early in the course of T1D was conducted by Carlsson et al. in 2015, in which 20 newly diagnosed (<3 weeks before enrollment) T1D patients were i.v.-injected with 2.1–3.6 × 10^6^ MSC from autologous bone marrow [106]. At the 1-year follow-up, a mixed meal tolerance test showed preserved or increased C-peptide response in MSC-treated patients compared to individuals receiving a placebo. Therefore, BM-MSC transplantation may prolong beta-cell function in recent-onset T1D patients [106]. Similarly, the safety and efficacy of the infusion of autologous BM-MSC was assessed in a trial of 21 recently diagnosed T1D patients [107]. BM-MSC were isolated, expanded, and delivered within 2 separate infusions at a high dose of 1 × 10^6^ cells/kg body weight. No adverse events related to transplantation were reported. Transplanted patients showed significantly lower numbers of hypoglycemic events and reduced HbA1c at 12 months post-transplantation, as well as significantly increased serum levels of anti-inflammatory cytokines IL-4 and IL-10 and decreased levels of pro-inflammatory TNFa. Unfortunately, no differences in fasting blood glucose levels, ability to lower glycemia following a meal, and requirements for exogenous insulin use were seen compared to participants receiving a placebo [107]. Hu et al. delivered allogeneic UC-MSC through one-time infusion into newly diagnosed T1D patients. Recipients showed elevated C-peptide levels starting at 6-months post-treatment, decreased HbA1c levels, and decreased postprandial plasma glucose levels 9-months after treatment. In addition, 8/15 patients required >50% decreased insulin dose, and 5/15 patients became insulin independent 12-months following injection, with independence persisting until the last follow-up at 24-months. These data demonstrate the capacity of MSC to induce partial recovery of islet function while also drawing attention to variability in MSC regenerative and immune-protective capacities based on donor heterogeneity and tissue source [108]. In 2016, Cai et al. conducted a clinical trial involving 42 patients with T1D who were randomized to receive co-transplantation of UC-MSC and autologous BM MNC via supraselective pancreatic artery canulation, or standard care (control). At one year, transplanted patients showed increased c-peptide area under the curve (AUC) and insulin AUC, as well as reduced fasted blood glucose levels, HbA_1C_, and exogenous insulin requirements. However, insulin independence was not achieved. Importantly, immunological parameters were also assessed after one year, which showed that transplanted patients had increased IL-10, decreased IFNg, and decreased ATP production by CD4^+^ T cells, indicating reduced T-cell activation [109]. Therapeutic strategies for T1D must address both the autoreactive host immune system and beta cell repair and regeneration. The dual immunomodulatory and regenerative secretory properties of MSC make them a promising therapy to dampen inflammation while enabling recovery of tissue function.

The first clinical study using portal vein infusion of islets alongside autologous BM-MSC was conducted by Wang et al. in 2018 [110]. Three subjects with chronic pancreatitis underwent total pancreatectomy and subsequently received islet and MSC co-transplantation [110]. During the 12 months following transplantation, patients showed improved glycemic control with less insulin and no direct adverse events associated with therapy [110]. Thus, the infusion of autologous BM-MSC during islet transplantation may have the potential to improve glycemic control and warrants further clinical studies (Figure 3).

These first clinical trials using MSC as a treatment for diabetes have shown some promise. However, there are still many important considerations before widespread therapeutic use is practical. Current trials are characterized by small sample sizes and heterogeneity in cell sources (BM- vs. UC-derived MSC), doses, and administration routes. The short follow-up durations reported also limit the generalizability and robustness of the trials. While some studies suggest improved glycemic control and decreased medication dependence, there are inconsistencies across trials, and the long-term durability of any observed therapeutic benefits is unknown. There is a critical need for larger, well-powered phase III trials with standardized protocols to establish therapeutic efficacy and optimal MSC regimens [111]. Using MSC in clinical trials for diabetes also involves important safety and regulatory considerations that must be carefully managed to ensure both patient protection and scientific integrity. To date, trials using MSC report a favorable safety profile, with most studies showing no serious adverse events; however, long-term safety data remain limited, and rare off-target effects have been raised as theoretical concerns [112]. Regulatory agencies require a robust demonstration of quality, safety, and efficacy from biological agents such as MSC before they can proceed in clinical trials or obtain market authorization [113]. A key regulatory challenge is the heterogeneity of MSC products, as cells derived from different tissues, donors, and culture conditions can vary widely in biological activity and clinical effects. Harmonizing protocols for cell sourcing, expansion, and delivery, and establishing internationally recognized quality standards are major ongoing efforts [114]. Regulators must also balance facilitating innovation with protecting trial participants by requiring rigorous trial design, long-term follow-up for delayed adverse events, and clear communication of risks. Ethical and practical considerations, such as informed consent and avoiding unproven commercial offerings outside regulated trials, are also important aspects of the regulatory landscape for MSC therapies.

Overall, while MSC therapy for diabetes shows potential, current clinical evidence remains preliminary. More rigorous and uniform clinical trials to substantiate MSC benefits beyond safety and positive short-term outcomes are required.

## 6. Conclusions

Given the urgent need for improved therapies for diabetes, all approaches to restoring insulin secretion in patients should be considered. With remarkable regenerative properties, MSC can be used to promote beta cell regeneration by awakening a developmental program in which pancreas-resident progenitor cells and pre-existing endocrine cell types are stimulated to form new beta cells. MSC can similarly support the function and survival of islets during transplantation in T1D patients. Comprehensive multiomic profiling studies are needed to fully understand the complexity of chemical messages sent by MSC and the response in pancreas-native progenitor cells. MSC licensing strategies can also be used to augment MSC islet regenerative capacity. Combining replacement pluripotent stem cell-derived islets with MSC or alternate islet supportive cell types [115,116] represents the future in islet replacement therapy. With further understanding of the mechanisms that control islet regeneration, we will be one step closer to re-establishing glucose homeostasis in diabetic patients, eliminating the burden of exogenous insulin suffered by T1D patients and their caregivers.

## Figures and Tables

**Table 1:** Summary of lineage tracing studies investigating pancreatic cell types and their conversion to beta cells in the adult mouse pancreas.

Reference	Traced Cell Type	Lineage Tracing Model	Model of Beta Cell Damage/Regeneration	Conversion to Beta Cells?
Dor et al. (2004) [53]	Beta cells	INS promoter-CreERT	Homeostasis	Yes
			70% Pancreatectomy (Px)	Yes
Zhao et al. (2021) [7]	All non-beta cells	INS2-Dre; Rosa26-iCre	Homeostasis	No
			Pregnancy	No
			STZ (150 mg/kg)	No
			70% Px	No
			Pancreatic ductal ligation (PDL)	No
			Diphtheria toxin	Yes; alpha and delta cells
Xiao et al. (2018) [54]	Alpha cells	GCG-CreERT	Alloxan + Pdx1/MafA viral delivery	Yes
Collombat et al. (2009) [55]	Alpha cells	GCG-Cre	STZ (200 mg/kg) + Pax4 overexpression in alpha cells	Yes
Courtney et al. (2013) [56]	Alpha cells	GCG-Cre	STZ (100–200 mg/kg) + Arx knockout in alpha cells	Yes
		GCG-rtTA/TetO-Cre		
Ben-Othman et al. (2017) [57]	Alpha cells	GCG-Cre	Long-term GABA treatment	Yes
	Ductal cells	Ngn3-CreERT		Yes
Thorel et al. (2010) [58]	Alpha cells	GCG-rtTA/TetO-Cre	Diphtheria toxin	Yes
van der Meulen et al. (2017) [59]	Alpha cells	GCG-CreERT2	Homeostasis	Yes
Pan et al. (2013) [60]	Acinar cells	Ptf1a-CreERT	PDL alone	Yes
			PDL + STZ (100 mg/kg/day × 2 days)	Yes
Zhou et al. (2008) [61]	Acinar cells	Cpa1-CreERT2	Ngn3/Pdx1/MafA viral delivery	Yes
Inada et al. (2008) [62]	Ductal cells	CAII-CreERT	PDL	Yes
Dirice et al. (2019) [6]	Ductal cells	CAII-CreERT	Pregnancy	Yes
El-Gohary et al. (2016) [63]	Ductal cells	Sox9-CreERT	50–60% Px + TGF-beta type II receptor upregulation	Yes
Kopp et al. (2011) [64]	Ductal cells	Sox9-CreERT2	Homeostasis	No
			PDL	No
Zhang et al. (2016) [65]	Ductal cells	Sox9-CreERT2	Alloxan+ long-term gastrin and EGF treatment	Yes
Solar et al. (2009) [66]	Ductal cells	Hnf1b-CreERT2	PDL	No
			Alloxan+ long-term gastrin and EGF treatment	No
Gribben et al. (2021) [67]	Ductal cells	Hnf1b-CreERT2	Homeostasis	Yes
		Ngn3-CreERT	AKITA mutant insulin 2	Yes
Al-Hasani et al. (2013) [68]	Ductal cells	Ngn3-CreERT	STZ (100–200 mg/kg) + Pax4 overexpression in alpha cells	Yes
		Hnf1b-CreERT2		Yes
Sancho et al. (2014) [69]	Ductal cells	CK19-CreERT	Fbw7 knockout in ductal cells	Yes
Chera et al. (2014) [70]	Delta cells	SST-Cre	Diphtheria toxin	Yes (Juvenile mice only)
Wang et al. (2020) [71]	Protein C receptor+ cells	Procr-CreERT2	Homeostasis	Yes
Rasiwala et al. (2025) [51]	Ductal and acinar cells	CK19-CreERT	STZ	Yes

## Data Availability

No new data were created or analyzed in this study.

## Abbreviations

The following abbreviations are used in this manuscript:

MSC	Multipotent stromal cells
T1D	Type 1 diabetes
T2D	Type 2 diabetes
BM	Bone Marrow
UC	Umbilical Cord
AD	Adipose
PANC	Pancreas
STZ	Streptozotocin
NOD/SCID	Non-obese diabetic/severe combined immunodeficiency
GFP	Green fluorescent protein
iPan	Intra-pancreatic
GSK3	Glycogen synthase kinase-3
CdM	Conditioned media
Px	Pancreatectomy
PDL	Pancreatic duct ligation
CK19	Cytokeratin 19
